# Photomechanical Wave-Driven Delivery of siRNAs Targeting Intermediate Filament Proteins Promotes Functional Recovery after Spinal Cord Injury in Rats

**DOI:** 10.1371/journal.pone.0051744

**Published:** 2012-12-14

**Authors:** Takahiro Ando, Shunichi Sato, Terushige Toyooka, Hiroaki Kobayashi, Hiroshi Nawashiro, Hiroshi Ashida, Minoru Obara

**Affiliations:** 1 Department of Electronics and Electrical Engineering, Keio University, Yokohama, Japan; 2 Division of Biomedical Information Sciences, National Defense Medical College Research Institute, Tokorozawa, Japan; 3 Department of Neurosurgery, National Defense Medical College, Tokorozawa, Japan; Zhejiang University School of Medicine, China

## Abstract

The formation of glial scars after spinal cord injury (SCI) is one of the factors inhibiting axonal regeneration. Glial scars are mainly composed of reactive astrocytes overexpressing intermediate filament (IF) proteins such as glial fibrillary acidic protein (GFAP) and vimentin. In the current study, we delivered small interfering RNAs (siRNAs) targeting these IF proteins to SCI model rats using photomechanical waves (PMWs), and examined the restoration of motor function in the rats. PMWs are generated by irradiating a light-absorbing material with 532-nm nanosecond laser pulses from a Q-switched Nd:YAG laser. PMWs can site-selectively increase the permeability of the cell membrane for molecular delivery. Rat spinal cord was injured using a weight-drop device and the siRNA(s) solutions were intrathecally injected into the vicinity of the exposed SCI, to which PMWs were applied. We first confirmed the substantial uptake of fluorescence-labeled siRNA by deep glial cells; then we delivered siRNAs targeting GFAP and vimentin into the lesion. The treatment led to a significant improvement in locomotive function from five days post-injury in rats that underwent PMW-mediated siRNA delivery. This was attributable to the moderate silencing of the IF proteins and the subsequent decrease in the cavity area in the injured spinal tissue.

## Introduction

The absence of spontaneous axonal regeneration after spinal cord injury (SCI) is attributed not only to the lack of neurotrophic factor support [1]–[3] but also to the presence of extracellular matrix inhibitors, such as chondroitin sulfate proteoglycans and myelin-associated inhibitors [4]–[7], and glial scar formation around the injury site [8]–[10]. Direct damage of spinal tissue is followed by inflammatory reactions in the vicinity of the lesion, being accompanied by the excessive appearance of reactive astrocytes. At the acute stages of SCI, the activated astrocytes are essential for blood-brain barrier repair, inflammation restriction, and protection of neurons and oligodendrocytes [11], [12]. On the other hand, they also show morphological modifications involving hyperplasia and hypertrophy [11], [13] and express various kinds of molecules associated with growth inhibitory properties [14]–[16]. One of the main molecular hallmarks of reactive astrocytes is the up-regulation of intermediate filament (IF) proteins such as glial fibrillary acidic protein (GFAP) and vimentin [17]–[19]. Over-expression of these IF proteins causes glial scar formation, resulting in a physical and biochemical barrier for axonal regeneration after SCI. In fact, double knockout mice lacking GFAP and vimentin showed lower levels of astroglial activity and astrocytic hypertrophy, and thus exhibited reduced scar formation after spinal cord hemisection [20].

Suppression of astroglial reactivity and scarring after SCI has been reported as a novel therapeutic strategy for reduction of glial scar formation [21]. It was reported that lentiviral delivery of small interfering RNAs (siRNAs) targeting GFAP and vimentin into primary cultured astrocytes improved neuronal survival and neurite outgrowth and prevented glial scarring [22]. Recently, using this strategy, we attempted to inhibit undesirable glial activity in SCI rats by cutting the meninges and intrathecally applying collagen-modified siRNAs targeting IF proteins, which resulted in substantial improvement in urinary function, though recovery of locomotive function was not significant [23]. Since the degree of locomotor recovery is highly correlated with the percentage of remaining normal nerve fibers in SCI rats [24], [25], it is necessary to efficiently and broadly reduce excessive astrogliosis and hence the cavitation area in the contused spinal tissue for achieving restoration of motor function based on such RNA interference (RNAi)-mediated treatment. In addition, site-specificity of gene transfection should be attained to avoid unnecessary gene uptake, since delivery to an intact site is often followed by negative immune responses. By conventional methods using viral vectors and chemical modification of delivered genes, it is difficult to spatially control the expression of a targeted gene with high accuracy.

As a method for site-selective gene delivery, laser-mediated gene transfer has received much attention because of the high spatial controllability of laser energy [26]. Lasers have mainly been used for direct irradiation of cells and tissues to perforate cell membranes. On the other hand, a laser is used to induce a defined impulsive pressure wave, known as photomechanical waves (PMWs) or laser-induced stress waves (LISWs) [27]–[36]. PMWs can increase the fluidity of cell membranes in tissue, enabling cellular uptake of exogenous genes such as plasmid DNA and siRNA. Genes coding for luciferase, enhanced green fluorescent protein (EGFP), β-galactosidase and human hepatocyte growth factor (hHGF) have been successfully delivered into rat skin *in vivo* by applying PMWs [27], [30]–[33], [36]. Plasmid DNAs coding for luciferase and EGFP have also been transferred into brains [28] and muscles of rodents [34]. More recently, reporter genes were delivered into uninjured rat spinal cords using PMWs and investigated transfection efficiency, distribution of gene expression and targeting characteristics [35]. The findings obtained in the study demonstrated the feasibility of PMW-based gene delivery into the targeted spinal tissue without functional damage [35]. Because pressure waves propagate through tissue much more efficiently than laser light itself, deep tissue can be treated with PMWs [31], [33]. These characteristics of PMWs potentially satisfy the requirements needed for siRNA therapy for SCI.

In the present work, we applied PMW-driven delivery of siRNAs to SCI model rats based on a strategy for axonal restoration by suppressing the over-expression of IF proteins. First, we transferred fluorescence-labeled siRNAs into SCI rats to investigate the distribution of siRNA uptake in the target tissue. We then delivered siRNAs targeting the IF proteins into injured spinal cords to promote the restoration of motor function in the rats. The reduction in the levels of the overproduced targeted proteins and glial scar formation were investigated as a function of time after injury.

## Materials and Methods

### Ethics Statement

All procedures used in this study were approved by the Committee on the Ethics of Animal Experiments at the National Defense Medical College (the permit numbers; 10041).

### Animal Experimental Procedures

We used female Sprague-Dawley rats (Japan SLC, Inc., Shizuoka, Japan) weighing 180–270 g in this work. The animals were housed one per cage after surgery and had free access to food and water except during periods of functional testing (see below). Before operation, they were anesthetized by intraperitoneal injection of pentobarbital sodium (50 mg/kg animal weight).

### Generation and Measurement of PMWs

As a laser-absorbing material (target), a 5-mm-diameter, 0.5-mm-thick black natural rubber disk was used. On top of the rubber sheet, a 1.0-mm-thick transparent polyethylene terephthalate sheet was bonded to confine laser-induced plasma, which can increase the peak pressure and pulse width of the PMWs generated [37]. PMWs were generated by irradiating the target with 532-nm Q-switched Nd:YAG laser pulses (Brilliant b, Quantel, Les Ulis Cedex, France; pulse width, 6 ns FWHM).

**Figure 1:**
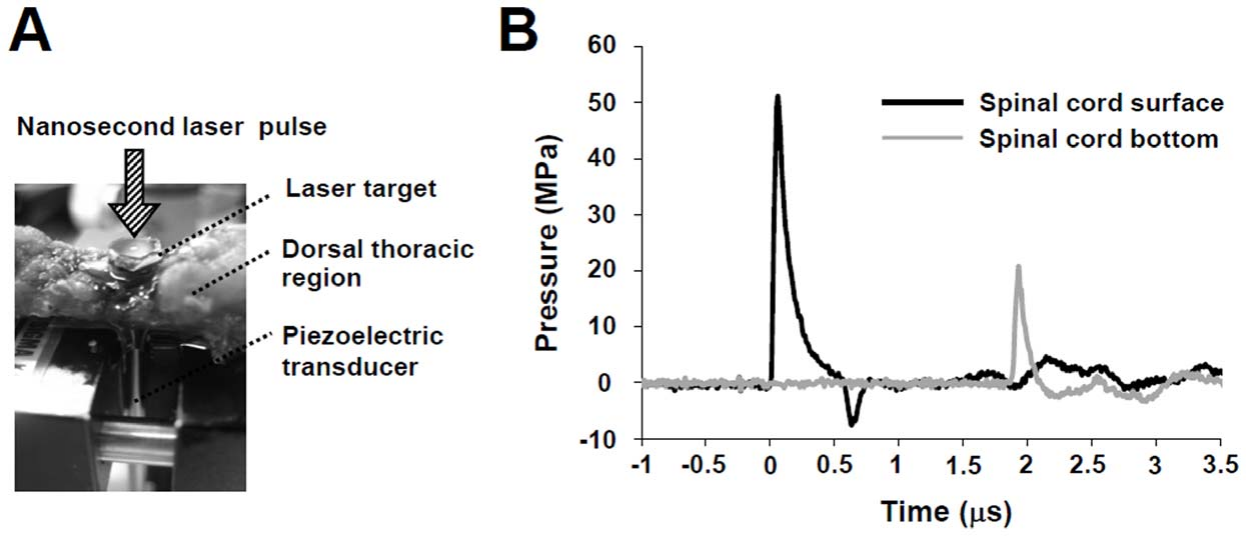
Measurement of the pressure characteristics of PMWs. (A) Schematic showing the setup for pressure measurement. (B) Temporal profiles of PMWs before and after propagation through the spinal column at a laser fluence of 0.3 J/cm^2^ with a 3-mm spot diameter. The thickness of the spinal matter was approximately 3 mm. Both profiles were dominated by positive pressure, suggesting low invasiveness of the pressure.

Pressure waveforms of PMWs were measured using a needle-type Pb(Zr, Ti)O_3_ hydrophone with a 1.0-mm-diameter sensitive area (HNR-1000, Onda, Sunnyvale, CA). To evaluate the propagation characteristics of PMWs through spinal tissue, the spinal cord was removed from a rat (n = 1). Pressure was measured before and after propagation through the spinal cord, for which the hydrophone was first placed directly under the target and then placed under the spinal cord (Fig. 1A). The thickness of the spinal cord was approximately 3 mm. Ultrasound conductive jelly (Echo Jelly, Aloka, Tokyo, Japan) was used to match the acoustic impedances of the hydrophone surface and the laser target or spinal tissue. The output signals from the hydrophone were recorded using a digital oscilloscope (TDS680B, Tektronix, Inc., Beaverton, OR; 1 GHz bandwidth).

### Spinal Cord Injury Model

The lamina of the tenth thoracic vertebra was surgically removed, and the spinal cord was exposed. A New York University (NYU) weight-drop device was used to make a severe spinal cord contusion; specifically, a 10-g weight was dropped from a height of 25 mm onto the spinal dura [38]–[40].

### Preparation of siRNA Solutions

Alexa-Fluor 488, 5′-labeled siRNA with control scrambled sequence was purchased from Qiagen (Valencia, CA); the duplex sequence was 5′-UUC UCC GAA CGU GUC ACG UdTdT-3′. An siRNA with a scrambled sequence (4390843, Ambion, Inc., Austin, TX) was used as a negative control. Synthetic siRNAs targeting rat GFAP (AM16704, ID: 49519) and vimentin (AM16704, ID: 189834) were obtained from Ambion, Inc. (Austin, TX); siRNAs targeting these IF proteins were selected based on the results of the *in vitro* study reported in the literature [23]. The 19-mer target sequence of the siRNAs targeting GFAP (NM_017009) and vimentin (NM_031140) were GGAACATCGTGGTAAAGAC targeting gene position 1236–1254 and GCTATGTGACCACATCCAC targeting gene position 166–184, respectively. To prevent rapid degradation of siRNAs in the presence of nuclease *in vivo* and to support the carrier activities of siRNAs, atelocollagen (Koken, Tokyo, Japan) was used in all experiments in this study; the collagen can stabilize gene fragments for at least one week and provide prolonged release of such fragments [41]–[45]. These characteristics have been reported to be effective for RNAi-based treatments that target slow-turnover proteins such as GFAP and vimentin [46]. For preparing complex of siRNAs and atelocollagen, equal volumes of atelocollagen and solution of siRNAs (20 µM) were mixed in a plastic tube by rotating for 20 min at 4°C, the final concentration of each siRNA being 10 µM.

### Delivery of siRNAs into Injured Spinal Tissue by Applying PMWs

Immediately after making a severe spinal cord contusion injury in a rat as described above, a 50-µl solution of Alexa-Fluor 488-labeled siRNA or scrambled siRNA or the same total volume of a mixture of siRNAs targeting GFAP (25 µl) and vimentin (25 µl) complexed with atelocollagen was intrathecally injected into several sites around the lesion through a Hamilton syringe with a 31-gauge needle (∼10-min administration time).

A laser-absorbing black rubber (target) was placed on the dura of the exposed injured spinal cord, for which ultrasound conductive jelly (Echo Jelly, Aloka, Tokyo, Japan) was used to match the acoustic impedances of the target and spinal tissue. PMWs were generated in the same manner as for pressure measurements. In all experiments with siRNA delivery, irradiation laser parameters were fixed; the laser fluence and pulse number were 0.3 J/cm^2^ and 10, respectively [35].

### Analysis of the Distribution of Fluorescence-labeled siRNAs

Distributions of fluorescence-labeled siRNA and GFAP expression in sagittal sections of injured spinal cords were examined at five days after trauma for the three groups: (1) SCI alone, (2) SCI plus siRNA injection, and (3) SCI plus siRNA injection followed by application of 10 pulses of PMW generated at a laser fluence of 0.3 J/cm^2^. Rats were anesthetized and sacrificed at five days after injury by transcardial perfusion with 150 ml of physiological saline, followed by further perfusion with 200 ml of 4% paraformaldehyde in physiological saline (n = 3, each group). Segments of the spinal cords centered on the injury site were removed and post-fixed in the same fixative overnight. Then, the tissues were frozen in an optimal cutting temperature (OCT) compound (Sakura Finetek USA Inc., Torrance, CA) and sectioned into 10-µm-thick slices using a cryostat microtome. The sections were incubated overnight at 4°C with rabbit polyclonal anti-GFAP (1∶3; N1506, Dako, Tokyo, Japan), and then were incubated with secondary antibody swine anti-rabbit IgG-TRITC (tetramethylrhodamine isothiocyanate) (1∶40 dilution) (R0156, Dako, Tokyo, Japan) for two hours at room temperature. We observed the distribution of fluorescence originating from Alexa-Fluor 488-labeled siRNA (green) and TRITC-labeled GFAP (red) in sagittal sections of the spinal cords centered on the injured site using a fluorescence microscope (Axiovert 200, Carl Zeiss, Thornwood, NY). For analyses if the distribution of fluorescence-labeled siRNAs as a function of tissue depth, three vertical lines were randomly placed on a typical green fluorescence digital image of the each injured tissue, and the number of pixels showing green fluorescence was counted along each line and integrated for every 300-µm depth section (n = 9, each group). Furthermore, the uptake of siRNAs into astrocytes was evaluated by merging the digital fluorescence images originating from Alexa-Fluor 488-labeled siRNA and TRITC-labeled GFAP, where the transfected cells appeared yellow due to colocalization. The total number of pixels showing a yellow color was counted in a randomly chosen 300 µm×300 µm area in the vicinity of the injury and was expressed as a function of the depth of the spinal cord (n = 5, each group).

### Western Blot Analysis

For western blot analysis, longitudinal 10-mm-long spinal segments centered on the injury were dissected at five days post-injury, and the tissues were immediately frozen with liquid nitrogen (n = 4, each group). To examine the effects of siRNA sequences on the expression of GFAP and vimentin, immunoblot analyses were performed under two conditions: (1) SCI alone (SCI group) and (2) scrambled siRNA injection after SCI (scrambled siRNA group). After being extracted from the excised spinal cords, the proteins were resolved with SDS-PAGE and transferred to polyvinylidene difluoride membranes (GE Healthcare, Buckinghamshire, UK). The membranes were immuno-blotted with antibodies against GFAP (ROI003, Cosmo Bio Co., Ltd., Tokyo), vimentin (sc-6260) and GAPDH (sc-32233, Santa Cruz Biotechnology, CA), followed by an incubation with horseradish peroxidase-conjugated secondary antibodies (Santa Cruz Biotechnology). After incubation with ECL reagent (GE Healthcare), chemiluminescence signals were photographed, and then the band intensities were quantitatively measured using an image analyzer.

In the following examinations, a mixture of siRNAs targeting GFAP and vimentin was delivered into the injured spinal cords of rats by applying PMWs. The experiments were performed under three different conditions: (1) SCI without any treatment (SCI group), (2) siRNA injection after SCI (siRNA group), and (3) siRNA injection and PMW application after SCI (PMW group). In the same manner as that described above, immunoblot analyses for GFAP and vimentin were performed under the three conditions. Control animals received sham surgery at five days before dissection (n = 4).

### Histological Analysis

In the same manner as described above, frozen sagittal serial sections (thickness of 10 µm) were prepared using a cryostat microtome after animal sacrifice at five days (n = 3, each group) or twenty-one days (n = 3, each group) after SCI. For diaminobenzidine (DAB) staining, after blocking of endogenous peroxidase with 3% hydrogen peroxide, the sections were incubated overnight at 4°C with rabbit polyclonal anti-GFAP (1∶3; N1506, Dako, Tokyo, Japan) or with mouse monoclonal anti-vimentin (1∶10; sc-6260, Santa Cruz Biotechnology, CA). After washing with Tween-PBS, the slices on slide glasses were incubated with secondary antibody linked with dextran polymer peroxidase complex for thirty minutes at room temperature. Peroxidase-based color development was performed using 3, 3-diaminobenzidine.

For evaluation of the morphological changes at twenty-one days after SCI, the adjacent sections were also stained with hematoxylin and eosin (HE). For a typical histological image at the lesion epicenter and adjacent sites (n = 3 for the same animal), cavitation areas in the longitudinal images of HE staining were manually outlined and quantified by image analysis (n = 9, each group).

### Anterograde Labeling

Regeneration of corticospinal tracts (CSTs) was assessed using an anterograde tracer (mini-ruby) injected into the motor cortices. Two weeks after SCI, animals were anesthetized with pentobarbital sodium and two holes of 1.0 mm in diameter were drilled in the cranium to expose both motor cortices (coordinates: 2 mm posterior to the bregma, 2 mm lateral to the bregma) (n = 4, each group) [47]–[50]. A 35-gauge needle of a Hamilton syringe was inserted through each hole to a depth of 1.0–1.5 mm and five injections were made for each side; each injection consisted of 1.0 µl of 10% tetramethylrhodamine biotinylated dextran (mini-ruby, Molecular Probes, Eugene, OR). The needle was retained in place for 2 minutes after completion of the injection to allow diffusion of the dextran. One week after the injection, the rats were perfused with 4% paraformaldehyde in physiological saline. In the same manner as that described above, frozen sections (thickness of 25 µm) were prepared using a cryostat microtome. The longitudinal sections at the lesion epicenter were examined under a fluorescence microscope (Axiovert 200, Carl Zeiss, Thornwood, NY).

### Evaluation of Locomotive Function

For the three groups of rats, the SCI group, siRNA group and PMW group, the motor function of the hind limbs was evaluated by open-field testing and scored based on the BBB scale [39], [40]; a score of 0 means no spontaneous movement while a score of 21 indicates normal locomotion (n = 12, each group). Assessment of the animals was performed before laminectomy and on days 1, 3, 5, 7, 10, 14, and 21 after the contused injury. The open-field consisted of a squared arena (45 cm×90 cm) with 20-cm-height walls. All rats received manual bladder expression before the open-field test to eliminate possible behavior differences due to bladder fullness. Experienced handlers placed the rat in the center of the open field and moved the rat back to the center if the rat stopped moving at the edge of the field. The open-field testing was recorded on video tapes during a 3-min observation period. Two examiners who performed the procedure were unaware of the groups to which the rats belonged.

### Statistical Analysis

Statistical analysis of the results of western blotting and cavity measurement was performed using one-way factorial analysis of variance (ANOVA) followed by Tukey’s *post hoc* test. Comparisons of the BBB scores of the three groups of rats were performed using two-way repeated ANOVA with Tukey’s *post hoc* test. A value of *P*<0.05 was regarded as statistically significant.

## Results

### Characteristics of PMWs used for Gene Transfection

To evaluate the propagation characteristics of PMWs through the spinal cord, temporal pressure profiles of PMWs before and after propagation through an extracted spinal cord were measured using a hydrophone under the conditions used for siRNA transfection (Fig. 1A). The waveforms both before and after propagation through the tissue were dominated by a compressive pressure component, suggesting low invasiveness of PMWs to the tissue (Fig. 1B) because biological tissues are generally much less susceptible to compressive stress than to tensile stress [51]. The maximum pressures at the spinal cord surface and the bottom were about 51 MPa and 20 MPa, respectively. The pulse widths of PMWs were as short as 80–100 ns (full width at half maximum, FWHM). Thus, the impulses of PMWs were relatively small despite the high peak pressures, which were 7.3 Pa·s and 1.8 Pa·s at the spinal cord surface and the bottom, respectively.

### Delivery of Fluorescence-labeled siRNAs into Injured Spinal Cords by PMWs

**Figure 2:**
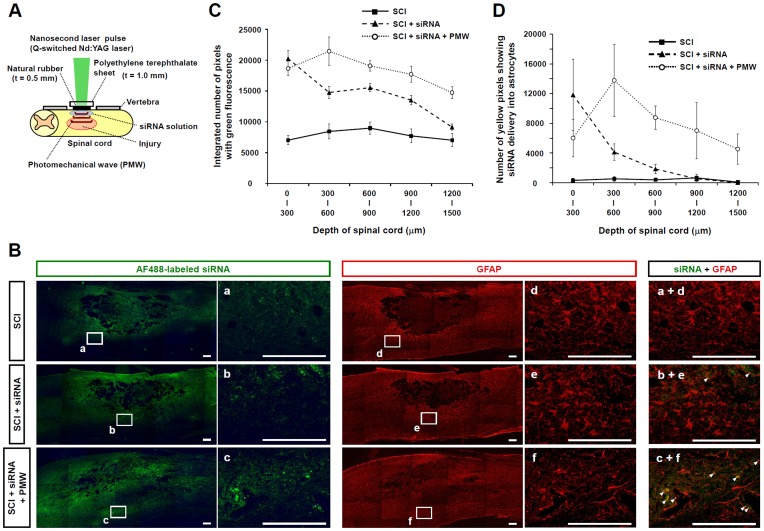
Delivery of fluorescence-labeled siRNA into injured spinal cords. (A) Experimental arrangement for PMW-based siRNA delivery into rat injured spinal cord. After laminectomy exposing a tenth dorsal vertebra followed by making a spinal contusion, the solution of siRNA was intrathecally injected around the lesion, to which PMWs were applied. (B) Distributions of fluorescence-labeled siRNA and GFAP expression in sagittal sections of injured spinal cords at five days after trauma for the three groups: SCI alone, SCI plus siRNA injection and SCI plus siRNA injection followed by application of 10 pulses of PMW generated at a laser fluence of 0.3 J/cm^2^. The enlarged and merged images show evident incorporation of siRNA into GFAP-positive astrocytes located in the anterior funiculus in the PMW application group (arrowheads). Scale bars represent 200 µm. (C) Depth dependence of fluorescence intensity from the fluorescence-labeled siRNA in injured spinal cords. Values are expressed as means ± S.E.M (n = 9, each group). The total number of pixels showing green fluorescence in the SCI alone group indicates the level of background autofluorescence. (D) Depth dependence of the number of pixels showing yellow, which indicates colocalization of siRNA with GFAP-positive astrocytes in injured spinal cords. Values are expressed as means ± S.E.M (n = 5, each group).

Fluorescence-labeled siRNAs were used to examine the distribution of siRNAs delivered by applying PMWs to injured spinal cord. Immediately after making a contusion in a rat spinal cord, Alexa-Fluor 488-labeled siRNA solution was intrathecally injected into the vicinity of the trauma. PMWs were then applied onto the dura mater (Fig. 2A). At five days after injury, the distributions of fluorescence originating from Alexa-Fluor 488-labeled siRNA (green) and TRITC-labeled GFAP (red) were observed in sagittal sections of the injured spinal cords under three different treatment conditions: no treatment (SCI alone) as a control, siRNA injection alone, and PMW application after siRNA injection. Low-intensity green fluorescence in the SCI alone indicates autofluorescence (Fig. 2B). High-intensity fluorescence was observed in the subsurface region of the spinal tissue with siRNA injection alone, while high-intensity fluorescence was spread over a much broader and deeper region of the SCI with PMW application after siRNA injection. Aggregation of GFAP was observed around the lesion site in all groups (Fig. 2B), indicating reactive astrogliosis in those regions. Magnified and overlaid fluorescence images of the injured spinal cords with PMW application revealed substantial incorporation of siRNA into GFAP-positive astrocytes (arrowheads, Fig. 2B).

Figure 2C shows the integrated numbers of pixels showing green fluorescence in the images as a function of the depth range of spinal tissues under the three conditions described above. The total number of pixels showing green fluorescence in the SCI alone condition, which ranged from 6000 to 10000, indicates the level of background autofluorescence. For the SCI with siRNA injection alone condition, the integrated number of pixels showing green fluorescence was much greater than the background level in the shallowest depth section (0–300 µm), but it rapidly decreased with increasing depth. In the PMW application group, on the other hand, a much higher fluorescence intensity level was observed across a wide range of depths of tissue (0–1500 µm), demonstrating the capability of PMWs for efficient siRNA delivery into the anterior side of the spinal cord. Figure 2D shows the integrated numbers of pixels with yellow fluorescence, which indicates colocalization of siRNA with GFAP-positive astrocytes, in the images as a function of the depth of the spinal cord. The results demonstrate that delivered siRNA was retained in the glial cells located in a deep region from 1000 µm to 1500 µm in the anterior funiculus at five days post-SCI.

### Effects of siRNA Sequences on Expression of GFAP and Vimentin

**Figure 3:**
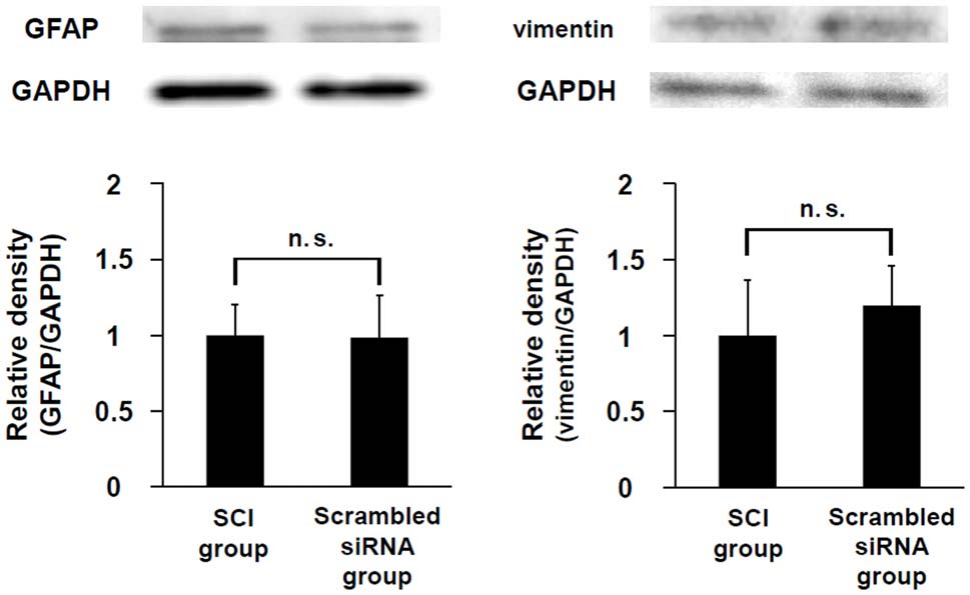
Effects of siRNA sequences on expression of GFAP and vimentin. Results of immunoblot analysis of GFAP and vimentin, which were extracted from 10-mm longitudinal spinal cord sections centered on the lesion site at five days after SCI. Results are expressed as means+S.E.M (n = 4, each group). No significant difference in the expression level of either IF protein was found between the SCI group and scrambled siRNA group. n.s. = not significant (*P*>0.05).

Figure 3 shows the expression levels of GFAP and vimentin in the two groups: (1) SCI alone (SCI group) and (2) scrambled siRNA injection after SCI (scrambled siRNA group). The expression levels of both IF proteins were similar in the two groups, indicating that the gene silencing effects in the following results were caused by the siRNA sequences.

### Delivery of siRNAs Targeting GFAP and Vimentin into Injured Spinal Cords using PMWs

**Figure 4:**
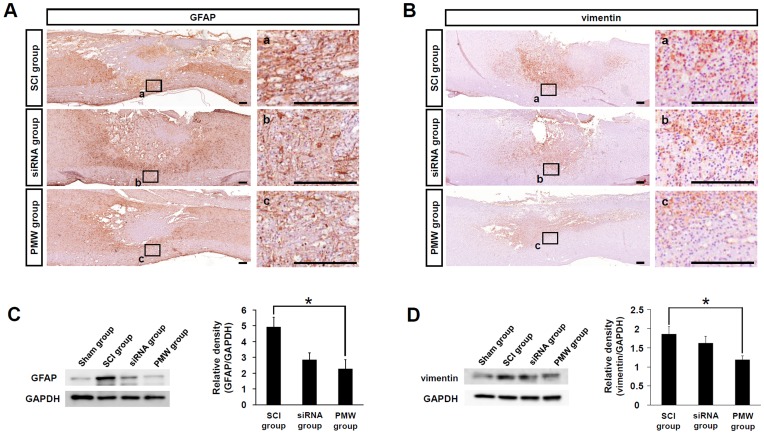
Effects of suppression of GFAP and vimentin at five days after trauma. The siRNAs targeting the IF proteins were delivered into the injured spinal cord using PMWs. Immunohistological images of longitudinal sections of injured spinal cords (A; GFAP, B; vimentin). Scale bars represent 200 µm. Results of western blot analysis of proteins extracted from 10-mm longitudinal spinal tissue segments centered on the injury site at five days after SCI (C; GFAP, D; vimentin). Relative expression levels of GFAP and vimentin in the SCI group, siRNA group and PMW group. Results are expressed as means+S.E.M (n = 4, each group). The expression levels of both GFAP and vimentin were standardized to those in intact spinal cords from sham-operated rats. The expression levels of these proteins increased remarkably after spinal injury, but significant suppression was observed when PMWs were applied after siRNA injection (*P<0.05).

On the basis of the results of the reporter siRNA delivery experiment described above, a mixture of siRNAs targeting GFAP and vimentin was delivered into the injured spinal cords of rats by applying PMWs. To examine the effects of transfection with these siRNAs, immunostaining for GFAP and vimentin was performed on sagittal sections of the spinal tissues at five days after SCI under three different conditions: (1) SCI group, (2) siRNA group, and (3) PMW group. In the SCI group, marked expressions of GFAP and vimentin were observed around the spinal contusion. Comparatively, the most silencing of GFAP was observed in the PMW group (Fig. 4A). Immunostaining for vimentin revealed that the expression of vimentin in the injured spinal tissue of the PMW group was obviously suppressed (Fig. 4B). To investigate this effect more quantitatively, immunoblot analyses were performed using spinal cords treated under the three conditions described above and intact spinal tissues after laminectomy as a sham-treatment control (Fig. 4C, 4D). In the intact spinal cord with sham treatment, the expression levels of both GFAP and vimentin were relatively low. After spinal injury, the expression levels of both IF proteins increased remarkably. Photographed chemiluminescence signals in western blot experiments were quantified by image analysis. When the protein expression levels in the sham-treated group were used for standardization, a 5-fold increase in GFAP expression level and a 2-fold increase in vimentin expression level were detected in the SCI alone group (Fig. 4C, 4D). When compared with the expression levels in the SCI group, moderate reductions in both GFAP and vimentin expression levels were observed in the siRNA group. Importantly, the application of PMWs after siRNA injection significantly reduced the expression levels of both proteins (*P*<0.05).

### Decreased Glial Scarring and Functional Recovery Following siRNA Delivery

**Figure 5:**
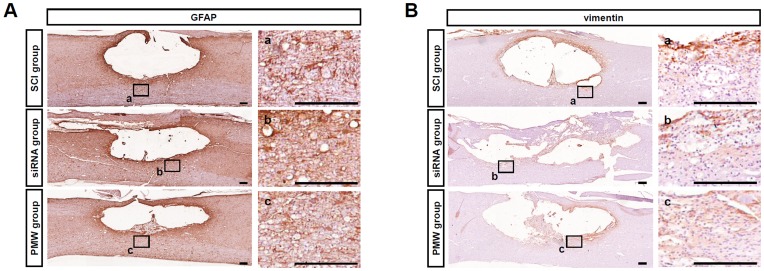
Immunohistological images of the spinal cord at three weeks after injury. Immunohistological images of longitudinal sections of injured spinal cords (A; GFAP, B; vimentin). Over-expressed GFAP and vimentin formed a major component of the glial scar; its astrocyte-rich structure would form a barrier to nerve fiber regeneration. Scale bars represent 200 µm.

**Figure 6:**
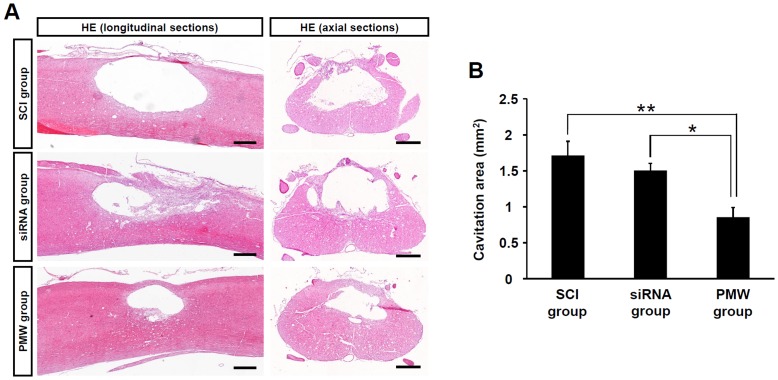
Decreased cavity area in the spinal cord at three weeks after injury. (A) Histological images of longitudinal and axial sections of injured spinal cords at three weeks after trauma. Scale bars represent 500 µm. (B) Results of quantitative analysis of the area of cavitary lesions in spinal cords on the basis of histological longitudinal images. Values are expressed as means ± S.E.M (n = 9, each group). The spinal cords of the PMW group showed comparatively smaller glial scars and the cavitation area was significantly reduced compared with those of the SCI group (**P<0.01) and the siRNA group (*P<0.05).

Immunohistological images of longitudinal sections of spinal cords at three weeks after injury showed aggregation of the IF proteins around the cavity and prominent formation of glial scars (Fig. 5A, 5B). The cavity areas in HE stained images were manually outlined and quantified by image analysis (Fig. 6A). Cavity areas in the PMW group were significantly reduced compared with those in the SCI group (*P*<0.01) and the siRNA group (*P*<0.05) (Fig. 6B). These observations show that PMW-based siRNA delivery reduces glial scar formation in injured spinal cords owing to the efficient suppression of over-expression of IF proteins.

**Figure 7:**
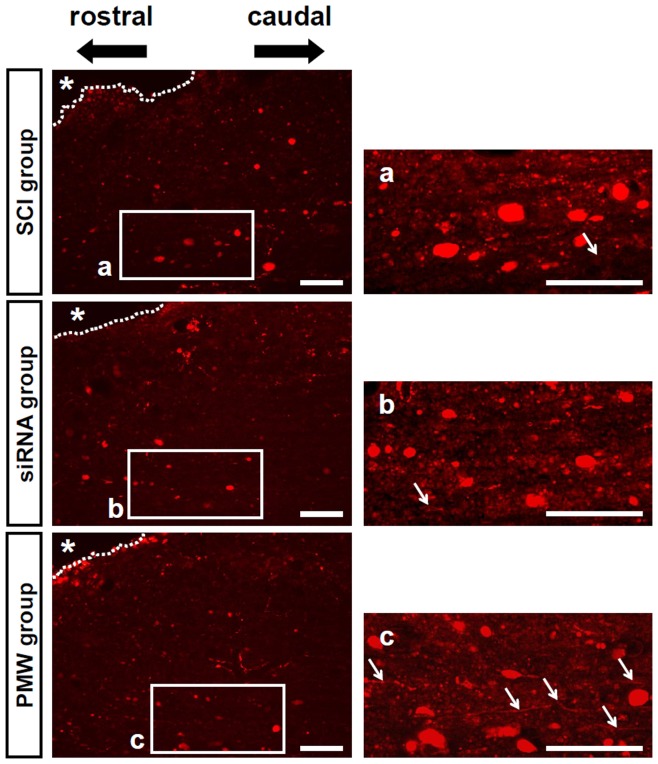
Anterograde tracer labeling of CST axons at three weeks after trauma. SCI rats in the PMW groups showed more retracting fibers in the region caudal to the trauma site than those in the SCI group and siRNA group (arrows). Asterisks depict cavity areas. Scale bars represent 100 µm.

Three weeks after PMW-based siRNA delivery, regeneration of the CSTs was assessed using an anterograde tracer (Fig. 7). It has been reported that retracting fibers in contusion SCI rat models circumvent the lesion through the spared ventral spinal tissue [52], [53]. This would be the reason for the labeled axons in the present results showing various directions around the lesion cavity. The SCI rats in the PMW group showed more sprouting CST axons in the region caudal to the trauma site than those in the SCI group and siRNA group.

**Figure 8:**
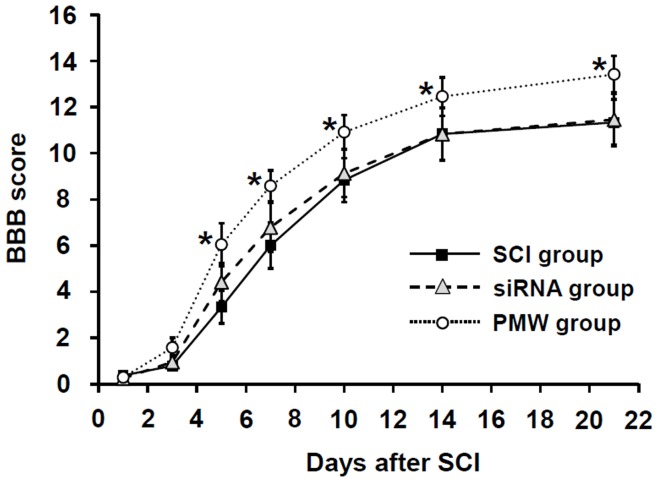
BBB scores after siRNA delivery. Results of motor function evaluation of hind limbs on the basis of open-field testing using the BBB scale at different time points after SCI. Values are expressed as means ± S.E.M (n = 12, each group). Asterisks mean significant differences compared with the values in the other two groups (*P<0.05).

The locomotive functions of the rats were assessed on the basis of Basso-Beattie-Bresnahan (BBB) scores [39], [40] as a function of days after SCI (Fig. 8). The weight drop-induced contusive SCI resulted in complete paralysis, and the animals suffered from long-lasting paralytic symptoms; the BBB scores at one day after SCI were 0 or 1 in all groups. Locomotive function gradually recovered and the scores almost reached a plateau within two weeks in all groups. From five days after SCI, a significant improvement in hindlimb motor function was observed in rats in the PMW group compared with those in the SCI group and the siRNA group (*P*<0.05); the average BBB scores at three weeks post-SCI were 11.3±1.0 in the SCI group, 11.5±1.1 in the siRNA group and 13.4±0.8 in the PMW group. This difference in BBB scores of 11 (no forelimb and hindlimb coordination) and 13 (frequent the coordination) is substantial and noteworthy.

## Discussion

The glial response to spinal injury results in recruitment of microglia, oligodendrocyte precursors, meningeal cells and astrocytes to the lesion site [8], [15], [54]. Among beneficial aspects, some of the astrocytes isolate the injury site and minimize the area of inflammation and cellular degeneration [11], [55]. On the other hand, most of the astrocytes in the injured area often become hypertrophic and adopt a reactive phenotype, releasing inhibitory extracellular matrix molecules such as chondroitin sulfate proteoglycans (CSPGs) [4], [5], [56] and ephrins [57]. The proliferation of reactive astrocytes is accompanied by an increase in the production of IF proteins such as GFAP and vimentin [18]–[20], [58], [59]. Remarkable reactive astrocytosis disturbs axonal regeneration, such that the traumatized spinal cord cannot recover properly, and permanent dysfunction remains as an inevitable result.

In the present study, we attempted to modulate the intrinsic behaviors of reactive astrocytes and hence to reduce glial scar formation. For this purpose, we delivered siRNAs targeting IF proteins into the injured spinal cords of rats *in vivo* by applying PMWs. Scar formation was efficiently reduced in the injured spinal tissue following application of PMWs after siRNA injection, demonstrating the validity of our method of delivery of siRNAs for treating SCI. To the best of our knowledge, this is the first report of an experimental demonstration of significant functional restoration in SCI rats achieved by delivering GFAP and vimentin siRNAs.

Although the detailed mechanism underlying PMW-based gene transfer is still under investigation, laser-induced impulsive pressures can increase the fluidity of the cell membrane into the target tissue, enabling cellular uptake of exogenous genes such as siRNAs and plasmid DNA [29], [60]–[65]. Previously, Tang *et al.* showed that PMW-mediated siRNA delivery induced posttranscriptional gene silencing in cultured plant cells [66], but to the authors’ knowledge, PMW-based *in vivo* gene silencing has not yet been reported.

PMWs are characterized by a unipolar compressive wave (Fig. 1B) and the formation of cavitation bubbles should therefore be limited, leading to minimally-invasive gene delivery. In general, gene delivery based on physical methods using naked plasmid is considered to be safe due to the lack of an immune response compared with methods using viral vectors [67], but physical energy might cause damage to tissue. For instance, Kondoh *et al.* stated in their report on gene delivery to the periventricular region in rats by electroporation that tissue damage caused by electrical shock was inevitable, and that electroporation is not suitable for gene delivery aimed toward neural regeneration [68]. Throughout the present experiments, no detrimental effects on rats were observed after PMW application, which is attributable to the low invasive nature of PMWs as described above.

Furthermore, PMWs can efficiently propagate through tissue with high directivity and limited energy attenuation owing to plane-wave characteristics [33]. Thus, neural cells located deep within the spinal tissue can interact with PMWs, enabling targeted delivery of siRNAs into deep spinal tissue. For gene transfer to deep tissue *in vivo*, ultrasound-based method can also be used. However, it has been reported that, with ultrasound microbubble-mediated transfection of spinal cords, the transfected cells were mainly meningeal cells at the dorsal surface of the spinal cord; no gene expression was observed in neurons or glial cells [69], [70]. This could be due to the limited distribution of microbubbles in the spinal cord. Moreover, although virus vectors have been widely used for gene delivery to the CNS, it is difficult to spatially control the region of gene expression because of their extreme transfection activities [71]–[75].

The transient maximum pressures of PMWs were measured to be about 51 MPa at the position of the spinal cord surface and 20 MPa under the spinal cord (Fig. 1B), indicating an explicit interaction of PMWs with deep glial cells. The peak pressure (20–51 MPa) and impulse (1.8–7.3 Pa·s) were considered to be high enough for PMW-mediated gene transfection [33], [35]. Because a siRNA solution was intrathecally injected to prevent damage to the spinal cord parenchyma, the diffusion and delivery of siRNAs into the parenchyma should be limited without PMW application. The results of fluorescence-labeled siRNA delivery showed that the depth of intense fluorescence from the siRNA was much increased by the application of PMWs; after subtracting the background autofluorescence, the integrated number of pixels showing green fluorescence in the spinal tissue following the application of PMWs was approximately 3.5 times larger than that with siRNA injection alone, achieving a depth of 1200–1500 µm (Fig. 2C). The colocalization of siRNA with GFAP-positive astrocytes indicated that glial cells were efficiently transfected by the fluorescence-labeled siRNA in the anterior funiculus at five days after SCI (Fig. 2B, 2D). Moreover, the results of immunostaining showed that the most evident reduction of GFAP and vimentin was achieved around the spinal contusion in the PMW group at five days after SCI (Fig. 4A, 4B, 4C, 4D). These observations are attributable to the capability of PMWs to deliver siRNAs into deeply located astrocytes in the spinal contusion.

As described above, astrocytes are activated after SCI with enlarged somas and intensive expression of IF proteins over time, and then a cystic cavity is formed in the region surrounded by the glial scar [21]. This neurodegenerative nature leads to a progressive increase in the size of the cavitation area [76], [77]. The results of immunohistological analysis at five days post-SCI showed that GFAP and vimentin were markedly up-regulated around the lesion in the SCI group, resulting in prominent formation of glial scars and cavities at three weeks after injury (Fig. 5A, 5B, 6A). For effective reduction of glial scar formation, it is necessary to deliver the relevant siRNAs into a broad region in the spinal contusion, and then to reduce astrogliosis. At five days post-SCI, the IF proteins were silenced especially in the white matter of the anterior horns in the PMW group. This shows that PMWs enhanced the uptake of siRNA into glial cells, especially those located in the ventral funiculus.

The anterograde tracing experiment showed numerous regenerating CST axons in the vicinity of the lesion in the PMW group (Fig. 7). This demonstrates that inhibition of excessive glial activity in the injured spinal tissue causes promotion of spontaneous axonal outgrowth, leading to functional recovery. The motor function of the lower limbs of rats in the PMW group was significantly improved from five days after injury (Fig. 8). These findings are consistent with the results of a previous study in which double mutant mice lacking GFAP and vimentin showed significant axonal regrowth of descending fibers of the corticospinal tract and ventral horn serotonergic tract, leading to an improved functional recovery after spinal cord hemisection [20].

To apply PMW-based siRNA therapy to larger animals with a thicker spinal column, deeper propagation of PMWs and broader distribution of therapeutic siRNAs are required. In our recent study, significant gene expression was observed in rat skin as a test tissue by applying PMWs that had to first propagate through 15-mm-thick tissue phantoms, demonstrating the capability of PMWs for cell permeabilization in tissues of ∼15 mm depth [33]. Catheter-based gene transfer can also be applied to large animals and humans because laser light can be delivered through an optical fiber [78]. For these applications, however, careful investigation is needed to determine the optimum PMW conditions.

In summary, we demonstrated the validity of PMW-mediated siRNA delivery for therapy for SCI. The application of PMWs promoted uptake of siRNAs targeting IF proteins into deep glial cells, enabling an evident reduction in the levels of the IF proteins. As a result, significant locomotive functional recovery was obtained in rats that underwent PMW application. PMW-mediated siRNA delivery is useful for safe and valid CNS therapy, having unique characteristics that cannot be realized by other methods.
